# High-performance scene classification in remote sensing imagery using a custom deep CNN architecture

**DOI:** 10.1038/s41598-025-34176-z

**Published:** 2026-02-09

**Authors:** Ahmed M. Abdelmonem, Mohamed Maher Ata, Abdelhamied A. Atey, A. A. Shaalan, Rania A. El-Sayed

**Affiliations:** 1 Department of Electronics and Communications Engineering, Zagazig University, Zagazig, 44519 Egypt; 2 School of Computational Sciences and Artificial Intelligence (CSAI), Zewail City of Science and Technology, October Gardens, 6th of October City, Giza, 12578 Egypt; 3 EIAS Data Science Lab, College of Computer and Information Sciences, Prince Sultan University, 11586 Riyadh, Saudi Arabia

**Keywords:** Remote sensing imagery, Scene classification, Deep convolutional neural network, Multi-class image, Recognition, Engineering, Aerospace engineering

## Abstract

This research presents a deep novel Convolutional Neural Network (CNN) architecture specifically designed for multi-class image categorization in remote sensing data. The proposed model is evaluated using both the NWPU-RESISC45 and UC Merced Land Use datasets, each containing 10 class categories: harbor, chaparral, tennis court, industrial area, parking lot, forest, beach, overpass, airplane, and baseball diamond. Extensive testing demonstrates that the proposed CNN architecture outperforms five popular pre-trained CNN models in terms of accuracy and efficiency. Quantitative results show that the proposed model achieves an accuracy of 0.9428 on the NWPU-RESISC45 dataset and 0.93 on the UC Merced dataset. The recall scores are 0.94 and 0.93, while precision values reach 0.95 and 0.94, respectively. Furthermore, the Intersection over Union (IoU) scores are 0.89 and 0.86, while the F1-scores are 0.94 and 0.93, confirming the robustness of the model across the datasets. In terms of computational efficiency, the model demonstrates competitive training times: 3,692 s for NWPU-RESISC45 (5,057 images across 10 classes) with GPU memory usage of 12.7 gigabytes (GB) and 559 s for the UC Merced dataset (722 images across 10 classes**).** To ensure and enhance the interpretability and explainability of the model’s predictions, two interpretability techniques were incorporated: Shapley Additive Explanations (SHAP) and Class Activation Mapping (CAM). **The key novelties of this manuscript include: **a hybrid CNN framework that not only advances classification performance but also incorporates explainability via SHAP and CAM, while maintaining efficient training times and strong generalization, making it a compelling solution for remote sensing image understanding. In general, the key novel aspects of our approach are: **Lightweight and efficient architecture:** Our proposed CNN design strikes a balance between performance and computational efficiency, making it highly suitable for real-time or resource-constrained environments without compromising accuracy. **Integrated interpretability:** By incorporating both SHAP (Shapley Additive explanations) and CAM (Class Activation Mapping), our framework delivers strong predictive performance. **Generalizability across datasets:** We demonstrate the model’s robustness and generalizability across two challenging and diverse datasets, confirming its adaptability to different domain scenarios.

## Introduction

Object detection in remote sensing imagery plays a vital role across various fields, including industrial monitoring, agricultural assessment, and military surveillance (D’Acremont et al., 2019)^1^. Accurate identification of land use patterns and surface features through satellite imagery is crucial for the effective regulation and analysis of both human activities and environmental changes (Lin & Wu, 2019)^2^.

 With the rapid expansion of high-resolution satellite imagery, deep learning techniques have become increasingly important for tasks such as image segmentation, target detection, object recognition, image enhancement, and data preprocessing (Liu et al., 2022)^3^. Among these techniques, Convolutional Neural Networks (CNNs) have emerged as the most dominant models for satellite image analysis, owing to their powerful hierarchical feature extraction capabilities (Ran et al., 2019)^4^.

These architectures enable detailed analysis of spatial and contextual information, significantly boosting object detection performance in remote sensing applications (Khan et al., 2020)^5^. However, proper model design and optimization are essential to fully harness the potential of CNNs in this domain. This involves tuning hyperparameters and adjusting network structures to enhance accuracy and efficiency.

In this paper, we present a novel CNN architecture specifically designed for multi-class object detection in remote sensing images. Our approach aims to reduce computational complexity and training time while maintaining high classification accuracy. The proposed model is evaluated using the UC Merced Land Use dataset and the NWPU-RESISC45 benchmark dataset, each covering ten scene categories. We systematically compare our model against five well-known pre-trained CNN architectures.

**Fig. 1:**
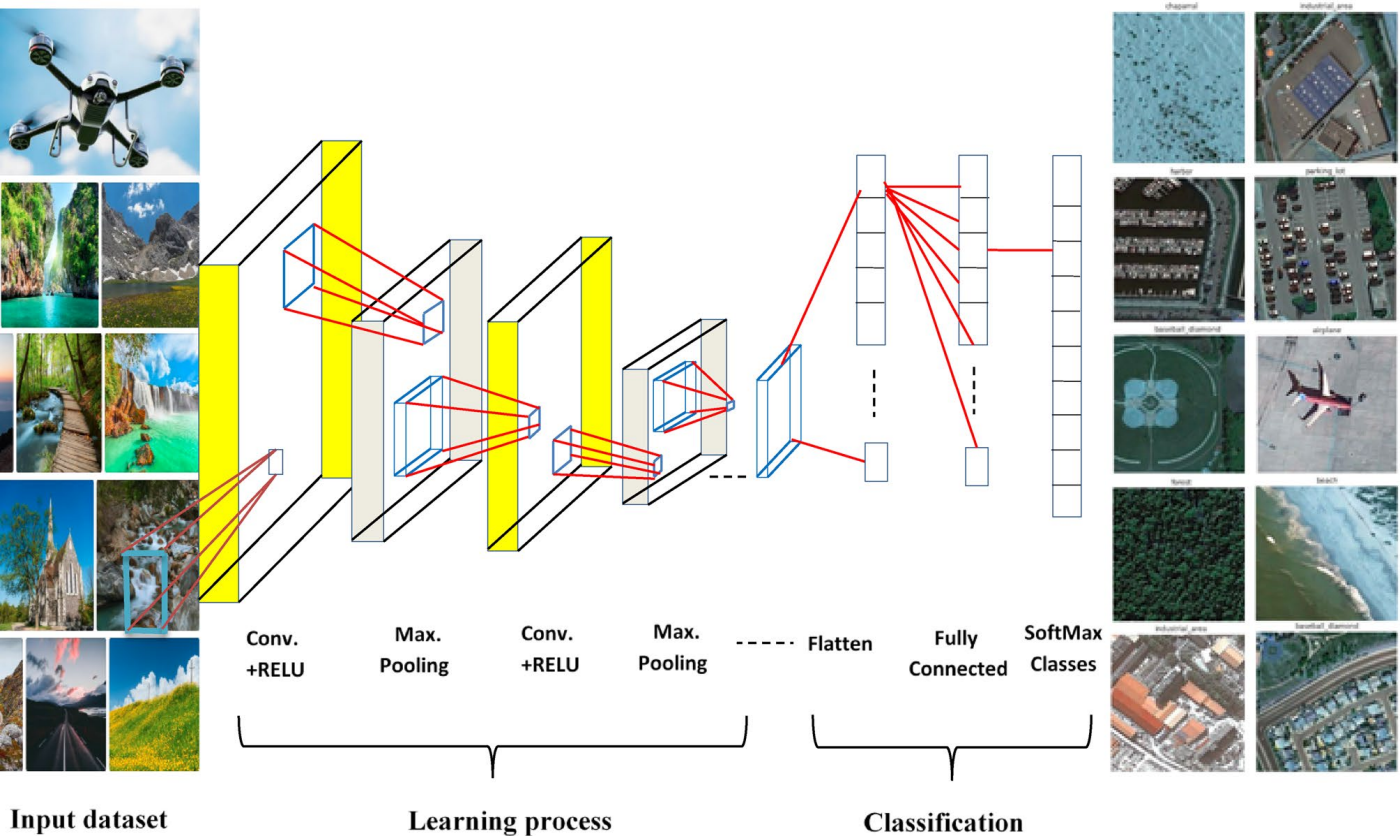
Key stages of the CNN structure model.

As shown in Fig. 1, a novel hybrid CNN architecture is presented for multi-class remote sensing scene classification. The figure illustrates the core structure of the proposed model and outlines the sequential pipeline starting with the input dataset, followed by convolutional layers with ReLU activation functions, max-pooling layers, and flattening operations. These are then connected to fully connected layers and, finally, to a SoftMax layer for multi-class classification.

The diagram effectively represents the CNN’s learning and classification process, emphasizing the hierarchical extraction and transformation of features throughout the network. It also incorporates interpretability mechanisms via SHAP and CAM. These tools provide essential insights into the model’s decision-making process by identifying key pixel-level contributions (via SHAP) and visualizing class-specific attention regions (via CAM). Together, they confirm that the model’s predictions are grounded in meaningful, human-interpretable features thereby enhancing trust, transparency, and potential real-world usability in remote sensing applications such as urban development, disaster response, and environmental monitoring.

According to the most important input features identified by SHAP, the model relies on three primary features, as shown in Table 1. To better understand the decision-making process of our Convolutional Neural Network (CNN) model, we conducted a SHAP (SHapley Additive exPlanations) analysis to identify the most influential features contributing to class predictions. For the **Airplane** class, runway length exhibited the highest SHAP values, indicating that the model strongly associates long, linear spatial patterns with the presence of aircraft. Additionally, the prominence of straight edges aligns with the geometric outlines of airplanes and runways, further validating the model’s reliance on meaningful structural cues.

In the **Baseball Diamond** class, green area size emerged as the most influential feature, reflecting the grassy field characteristic of typical baseball diamonds. The model also responded to structural compactness, which likely corresponds to the tight geometric layout of bases and perimeter fencing.

For the **Harbor** class, water pixels were the dominant factor, demonstrating the model’s sensitivity to large water bodies commonly found in port areas. Furthermore, the significance of object density suggests that the model effectively captures clustered elements such as docked ships and harbor infrastructure.

Overall, features like runway length and water coverage consistently ranked among the most important across classes, highlighting the model’s ability to learn semantically meaningful and spatially coherent patterns. These findings confirm that the model’s internal representations align well with human-interpretable features and underscore the value of SHAP in validating CNN behavior for remote sensing scene classification.

The development of the proposed model involves three main stages:

1. Data Preparation: Collecting and preprocessing remote sensing datasets.
2. Model Development: Designing and training a novel CNN architecture optimized for scene classification.
3. Performance Evaluation: Comparing the proposed model with multiple pre-trained CNNs based on classification accuracy and computational efficiency.

This paper also addresses key considerations for optimizing CNN models for remote sensing data analysis:

1. Custom CNN Design: A reliable CNN model with carefully crafted architecture and optimized hyperparameters is proposed for enhanced classification performance.
2. Evaluation of Pre-Trained Models: The study investigates the performance of several widely used pre-trained CNNs in the context of remote sensing imagery.
3. Comprehensive Comparison: A detailed comparative analysis is conducted between the proposed CNN and five pre-trained models to highlight performance gains.

**Table 1:** Most input features models rely on, according to SHAP.

Scene Class	Most Influential Features (High SHAP Value)
Airplane	Runway length, Straight edges
Baseball Diamond	Green area size, Structure compactness
Harbor	Water pixels, Object density

The remainder of this paper is organized as follows: Sect. “Literature review” summarizes related work in remote sensing and CNN-based methods, Sect. “Approach preprocessing” outlines the data collection and preprocessing strategy, Sect. “The Proposed CNN model approach” presents the architectural details and training strategy of the proposed model, Sect. “Experimental results and discussion” reports the results and comparative analysis, and finally, the paper is concluded with insights and potential future directions.

## Literature review

### Classification

Pallavi Ranjan et al. (2025) introduced a novel semi-supervised framework that synergizes autoencoders, generative adversarial networks, and zero-shot learning. This semi-supervised approach significantly improves feature extraction and data augmentation by harnessing the power of generative adversarial networks built upon autoencoders, ultimately enhancing classification accuracy. It further pushes the boundaries beyond traditional methods by enabling zero-shot learning, allowing the model to classify unseen data from classes not present in the training set^6^. Pallavi Ranjan et al. (2024) presented a novel approach for generating high-quality synthetic hyperspectral data cubes using an advanced Generative Adversarial Network (GAN) integrated with the Wasserstein loss and gradient penalty phenomenon (WGAN-GP). This approach aims to augment real-world data, mitigating the scarcity of labeled samples that has long been a bottleneck in hyperspectral image analysis and classification. To fully leverage both the synthetic and real data, we introduce a novel Convolutional LSTM classifier designed to process the intricate spatial and spectral correlations inherent in hyperspectral data. This classifier excels in modeling multi-dimensional relationships within the data, effectively capturing long-term dependencies and improving feature extraction and classification accuracy. The performance of our proposed model, termed 3D-ACWGAN-ConvLSTM, is rigorously validated using benchmark hyperspectral datasets, demonstrating its effectiveness in augmenting real-world data and enhancing classification performance^7^. Rohith and Kumar (2022) developed a 13-layer CNN architecture to classify raw remote sensing images from the National Remote Sensing Centre (NRSC) dataset^8^.

Unnikrishnan et al. (2019) optimized well-known architectures such as VGG, ConvNet, and AlexNet by reducing layers and employing two-band input data, allowing for efficient classification of satellite images into multiple categories^9^. Pallavi Ranjan et al. (2025) highlighted the transformative potential of combining hyperspectral imaging (HSI) with advanced autoencoder architectures to address critical challenges in high-dimensional data analysis. By leveraging the powerful feature extraction and dimensionality reduction capabilities of autoencoders, hyperspectral data can be effectively processed to enhance classification accuracy, reduce noise, and uncover subtle spectral-spatial patterns. The synergy between HSI and deep learning models, particularly autoencoders, opens new avenues for real-time, accurate, and scalable remote sensing applications. Future research should continue to explore hybrid frameworks and semi-supervised strategies that can further improve performance, especially in scenarios with limited labeled data. As imaging technologies and computational models evolve, this integration is poised to play a key role in next-generation Earth observation and environmental monitoring systems^10^. Ma et al. (2021) proposed SceneNet, a neural architecture discovered through multi-objective evolutionary optimization. This approach facilitated better extraction of hierarchical information from satellite images^11^. Dong et al. (2020) integrated CNN with Random Forest (RF) classifiers to improve the classification accuracy of extremely high-resolution satellite images through hybrid feature selection^12^. Cheng et al. (2020) addressed challenges in satellite image classification and proposed techniques based on auto-encoders, CNNs for object detection, and Generative Adversarial Networks (GANs)^13^. Xu et al. (2021) combined Recurrent Neural Networks (RNNs) with Random Forests to perform object and pixel-level land classification using remote sensing data^14^. Liang et al. (2020) proposed a two-stream classification system that combines CNN and Graph Convolutional Networks (GCN) to extract both global visual and spatial object-based features, enhancing feature discrimination^15^. Li et al. (2019) developed and compared four deep neural network-based classification frameworks (CapsNet, SMDTR-CNN, SMDTR-CapsNet, and CNN) tailored for urban environment classification^16^. Kumar et al. (2021) evaluated 16 CNN architectures pre-trained on ImageNet and demonstrated how fine-tuning these models can improve classification accuracy in high-resolution satellite images, thereby reducing the dependency on large datasets^17^.

### Detection

Cui et al. (2021) introduced a dual-channel deep learning (DCDL) model that uses multiscale convolution residual networks to identify three classes in the NWPU-RESISC45 dataset by incorporating both local and global features^18^. Song et al. (2025) introduce Vision transformers (ViT) detectors excel in processing natural images. A novel Faster R-CNN-based method, referred to as QAGA-Net, is proposed to substantially improve the performance of Vision Transformer (ViT) detectors in remote sensing image (RSI) recognition. QAGA-Net demonstrates an improvement in mean Average Precision (mAP) by 2.1% and 2.6% on the challenging DIOR20 dataset when compared to leading CNN-based and ViT-based detectors, respectively^19^.

Zhang et al. (2022) proposed an aircraft detection system based on modified Faster R-CNN (MFRC), incorporating K-means clustering, modified pooling in VGG16, and Soft-NMS for enhanced performance^20^. Sharma et al. (2021) presented YOLOrs, a CNN framework optimized for object detection in multimodal remote sensing images, particularly for vehicle detection^21^. Pallavi Ranjan et al. (2023) demonstrated the effectiveness of semi-supervised learning (SSL) techniques in addressing the challenges of hyperspectral image (HSI) classification, particularly under limited labeled data scenarios. By leveraging both labeled and abundant unlabeled data, the proposed SSL framework significantly enhances the stratification accuracy of hyperspectral images while reducing the dependency on costly manual annotations. The results affirm that semi-supervised models not only bridge the gap between supervised and unsupervised methods but also offer robust generalization across diverse spectral-spatial contexts. Future work will explore more advanced graph-based and contrastive SSL approaches, as well as domain adaptation techniques, to further elevate performance in real-world remote sensing applications^22^. Karnick et al. (2022) used the COWC dataset and proposed a Multi-Scale Swift Detection System (MSSDS) using a modified YOLO architecture, YOLT, to locate cars in variable-sized test images^23^. Büyükkanber et al. (2024) explore the impact of various data augmentation techniques and hyperparameter configurations on the training performance of the single-stage YOLOv5 deep learning model, applied to the DIOR remote sensing dataset for multiclass object detection. Among the experiments conducted, the approach achieved a mean Average Precision (mAP) score of 0.628^24^.He et al. (2025) used the YOLOv11 model to train and detect ground object targets in high-resolution remote sensing imagery, aiming to evaluate its effectiveness in improving detection accuracy and computational efficiency. The model was trained on a dataset of 70,389 samples spanning 20 different object categories. After 496 training epochs, the loss functions Box_Loss, Cls_Loss, and DFL_Loss showed rapid convergence, indicating successful optimization in object localization, classification, and fine-grained feature refinement. Evaluation metrics showed strong performance, with a precision of 0.8861, a recall of 0.8563, mAP 0.50 of 0.8920, mAP of 0.50:0.95 of 0.8646, and an F1-score of 0.8709, reflecting the model’s high accuracy and robustness in complex detection scenarios. Additionally, 80% of test samples had confidence scores above 85%, further confirming YOLOv11’s reliability in multiclass, multi-object detection tasks^25^.

Darehnaei et al. (2022) introduced Swarm Intelligence-Ensemble Deep Transfer Learning (SI-EDTL) that integrates Faster R-CNN with multiple transfer models to detect four classes of vehicles using UAV data^26^. Chen et al. (2022) proposed Domain Adaptation Faster R-CNN (DA-Faster R-CNN) to improve aircraft detection in low-quality remote sensing images using transfer learning strategies^27^. Zhu et al. (2024) reviewed multi-class change detection techniques in satellite remote sensing imagery. The paper focuses on methods that address the identification of changes across different classes over time, contributing to applications in environmental monitoring and urban planning^28^.

### Segmentation

Pang and Gao (2022) introduced MAGC-Net, a segmentation architecture that incorporates multi-head attention and Conv-LSTM for pixel-wise classification of ocean satellite images. The approach reduces redundant features and improves fusion quality^29^. Gui et al. (2024) introduced that recent developments in object detection techniques, alongside their closely related counterpart—instance segmentation—have made notable strides in the deep learning field. The fusion of these methodologies allows for more comprehensive approaches to handling data across different scales and modalities, such as optical imagery, synthetic aperture radar (SAR) images, and digital surface models (DSM). This integration enhances the ability to analyze and interpret complex datasets, offering new opportunities for precise object recognition and segmentation in various remote sensing applications^30^.

### Summary and categorization

Based on the reviewed literature, remote sensing image analysis techniques can be broadly categorized as follows:

1. **Objects**: Focused on single object vs. multi-object detection.
2. **Image Acquisition**: Using drones versus satellites.
3. **Image Resolution**: Applications with low-resolution vs. high-resolution imagery.
4. **Application Task**: Classification, detection, and segmentation.

Table 2 summarizes the most recent research efforts in remote sensing image classification, particularly emphasizing studies addressing the challenge of identifying multiple objects. The table outlines the techniques, datasets, and innovations used, offering a comprehensive overview of current advancements in the field.

**Table 2:** The most research efforts in remote sensing image classification by CNN models.

References	Techniques	Applications	Strength	Challenges
Özyurt (2020)	CNN—Relief—SVM	Feature selection	High accuracy	Complexity and time
Zhao et al. (2019)	Pre-trained CNN models	Classification	Comparison of feature representation methods	No new architecture
Rohith and Kumar (2022)	Thirteen-layer deep CNN model	Classification	Layering structure	Low accuracy
Unnikrishnan et al. (2019)	AlexNet, ConvNet, VGG	Classification	Hyper-tuning the pre trained networks	Lack of performance indicators
Priya and Vani (2019)	Inception-v3	Classification	High accuracy	Single class and small dataset
Ma et al. (2021)	SceneNet	Classification	Multi-objective optimization	Very Large training time
Dong et al. (2020)	CNN-RF	Classification	Fusion of CNN and RF for feature extraction and classification	A large number of epochs
Cheng et al. (2020)	Published classification techniques	Classification	Comprehensive comparison of classification methods	traditional architecture
Xu et al. (2021)	RNN-RF	Classification	Classification technique	Small dataset size
Liang et al. (2020)	CNN-GCN	Classification	Features fusion	Complexity
Li et al. (2019)	SMDTR-CNN	Classification	New layering structure (Capsule)	Large training time
Kumar et al. (2021)	16 Pre-trained CNN models	Classification	Parameters tuning	traditional architecture
Cui et al. (2021)	DCDL	Recognition	Mining global and local features simultaneously	Recognition is for a single class
Zhu et al. (2020)	FB-FRC	Detection	Features fusion strategy	Complexity
Zhang et al. (2022)	Faster RNN	Detection	Boundary box optimization	Single object detection
Sharma et al. (2021)	YOLOrs	Detection	Detect objects at several scales	Complexity and processing time
Karim et al. (2019)	RCNN	Detection	Impact of image rescaling on detection	No new architecture
Karnick et al., (2022)	YOLT	Detection	Detect objects at several scales	Complexity
Napiorkowska et al. (2018)	VGG-8	Detection	High accuracy	Single object detection
Feng et al. (2019)	Modified RPN	Detection	Layering structure	Many iterations and lack of indicators
Darehnaei et al. (2022)	SI-EDTL	Detection	Combined 3 pre-trained models with 5 classifiers	Complexity and processing time
Chen et al. (2022)	DA faster R-CNN	Detection	Fast iteration time and low brightness domain adaptation	Single object detection
Zalpour et al. (2020)	Improved faster R-CNN with SVM	Detection	Small processing time	Single class
Pang and Gao (2022)	MAGCNet—ConvLSTM	Segmentation	Feature extraction technique	Single object segmentation
Diakogiannis et al. (2020)	ResUNet-a	Semantic segmentation	Multiple object segmentation	Only used with high resolution images

## Approach preprocessing

Preprocessing constitutes a foundational step in preparing remote sensing imagery for deep learning applications, particularly in tasks such as scene classification, where spatial and spectral consistency significantly affect model performance. In this study, a multi-stage preprocessing pipeline was designed to optimize input compatibility with CNN architectures, enhance the diversity of training samples, and mitigate risks of overfitting and data imbalance.

The first step involved resizing images to ensure that all input images conformed to the structural requirements of the CNN models. Images were resized to 256 × 256 × 3 for the proposed pyramidal CNN model, aligning with its input layer dimensions. For pre-trained models such as VGG16, ResNet50, and Xception, standard input sizes were used (224 × 224 × 3 or 299 × 299 × 3), as specified in their original configurations. This resizing ensured uniformity in spatial dimensions, minimized computational overhead, and preserved feature scale compatibility across layers.

Data normalization is addressed through uniform image resizing rather than explicit pixel intensity scaling. All input images are resized to dimensions of 256 × 256 pixels to ensure compatibility with the model architecture. To further improve generalization and reduce overfitting, data augmentation techniques were systematically applied across the training set. These included horizontal and vertical flipping, which simulate mirrored views of the same scene and enable the model to learn spatial invariance.

Additionally, random rotations within the range of 0° to 180° were introduced to mimic various orientations of objects in satellite imagery, such as roads, rooftops, and coastlines. A zoom-in range of 0.0 was specified, indicating no zooming was applied in this version of the augmentation. However, in a separate experiment, brightness augmentation was included, using a brightness variation range of [0.1 to 2.0], to simulate changing illumination conditions across seasons or times of day, a common challenge in remote sensing data. These augmentations not only increase the effective size of the training dataset but also introduce controlled variability, allowing the model to become more robust to real-world inconsistencies.

All preprocessing steps, including acquisition, resizing, normalization, augmentation, and optimization (which will be shown as subtitled) were implemented using Python-based libraries (like Keras, TensorFlow, or OpenCV), and were applied uniformly across both the proposed model and the benchmark pre-trained CNNs. This consistency ensured fairness in experimental comparison and prevented data leakage or augmentation bias. The overall preprocessing strategy was crucial in enhancing feature discriminability, reducing noise, and ensuring that the input data fed into the model were standardized, diverse, and representative of real-world remote sensing conditions.

### Image acquisition

**Fig. 2:**
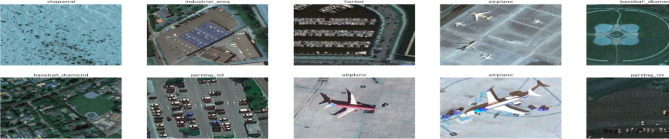
Examples from the NWPU-RESISC45 dataset.

**Fig. 3:**
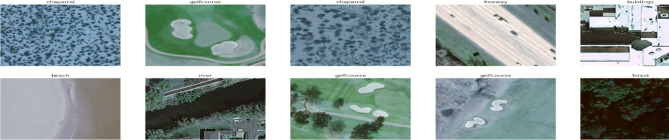
Examples from the UC Merced dataset.

This study utilizes two well-established remote sensing datasets, summarized in Table 3:

- **NWPU-RESISC45**: Developed by Northwestern Polytechnical University, this dataset comprises 31,500 images categorized into 45 distinct scene classes, with 700 images per class. Each image is in RGB format with a resolution of 256 × 256 pixels (Cheng et al., 2017)^31^.
- **UC Merced Land Use Dataset**: This dataset includes aerial images representing 21 land-use categories. Each class consists of 100 RGB images, each with a resolution of 256 × 256 pixels (Yang & Newsam, 2010)^32^. Figures 2 and 3 provide representative samples from the two datasets.

**Table 3:** Overview of the two datasets.

Dataset name	Classes number	No. of images per class	Image size
NWPU-RESISC45	45	700	256 × 256 × 3
UC Merced	21	100	256 × 256 × 3

### Image preprocessing

**Fig. 4:**
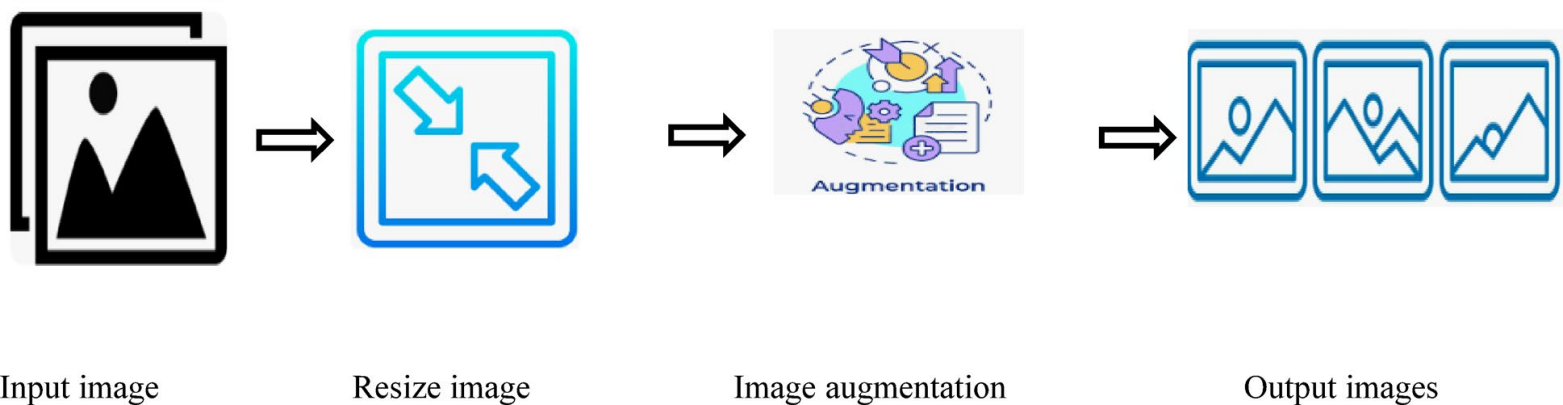
Preprocessing flow chart.

Preprocessing plays a vital role in enhancing the quality of data fed into deep learning models, especially in tasks like object detection and classification. Two primary preprocessing techniques image resizing and image augmentation were applied in this study (Marastoni et al., 2021; Kodali & Dhanekula, 2021)^33^. The overall preprocessing pipeline is illustrated in Fig. 4.

### Image resizing

Before model training, all input images were spatially resized to 256 × 256 pixels to maintain consistent input dimensions across the dataset; no normalization or rescaling of pixel intensity values (i.e., values remained in the original 0–255 range) was applied. Image resizing is employed to align the input dimensions with the structural requirements of the utilized CNN architectures. This preprocessing step not only ensures input compatibility but also helps to decrease computational overhead and may contribute to enhanced model accuracy (Pathak & Raju, 2022)^34^. All images were resized using Python-based functions to match the input dimensions of the corresponding pre-trained CNN architectures (Vyas et al., 2022)^35^.

### Image augmentation

To increase the diversity and volume of training data, data augmentation techniques were implemented. This approach helps mitigate overfitting by creating new training samples via transformations of existing images (Chlap et al., 2021; Khalifa et al., 2022)^36^. These transformations enabled the model to generalize better across varied environmental scenes.

The following augmentation techniques were applied:

- Horizontal and vertical flipping.
- Random rotation (0°–180°).
- Zooming (with 0.0% zoom factor).

### Model optimization

Optimizers adjust network weights during training to minimize loss and improve model accuracy. In this study, the **Adam** optimizer was employed with the following configuration:

- Initial learning rate: 0.00025.
- Learning rate scheduler: ReduceLROnPlateau strategy with a reduction factor of 0.25.
- Patience: 4 epochs (i.e., learning rate is reduced if accuracy does not improve for 4 consecutive epochs).
- Minimum learning rate: 1 × 10⁻⁶ (Manickam et al., 2021; Kingma & Ba, 2015)^37,38^.

### Pre-trained CNN architectures

Several pre-trained Convolutional Neural Networks (CNNs) were utilized (it is used only five pre-trained CNN from these pretrained models) to benchmark the performance of the proposed model. These include:

- **VGG16**: Composed of 13 convolutional and 3 fully connected layers, totaling 41 layers. Input size: 224 × 224 × 3 (Ye et al., 2021)^39^.
- **VGG19**: Comprises 16 convolutional and 3 fully connected layers, also totaling 41 layers. Input size: 224 × 224 × 3 (Li et al., 2020)^40^.
- **AlexNet**: Contains 5 convolutions, 3 pooling, and 3 fully connected layers. Input size: 224 × 224 × 3 (Dhillon & Verma, 2020)^41^.
- **MobileNet**: Features depthwise separable convolutions with 28 layers. Total parameters: ~4.2 M. Input size: 224 × 224 × 3 (Hou et al., 2020)^42^.
- **DenseNet**: Utilizes dense connectivity, where each layer is connected to every other layer. Input size: 224 × 224 × 3 (Zhai et al., 2020)^43^.
- **LeNet**: One of the earliest CNNs, composed of two convolutional layers followed by three fully connected layers. Input size: 32 × 32 × 3.
- **Xception**: A 71-layer deep CNN with depthwise separable convolutions. Input size: 299 × 299 × 3 (Jie et al., 2020)^44^.
- **Inception-V3**: Includes inception modules with symmetric and asymmetric architectures. Input size: 299 × 299 × 3 (Kumthekar & Reddy, 2021)^45^.

These pre-trained models serve as baselines for evaluating the proposed approach’s classification performance.

## The proposed CNN model approach

**Fig. 5:**
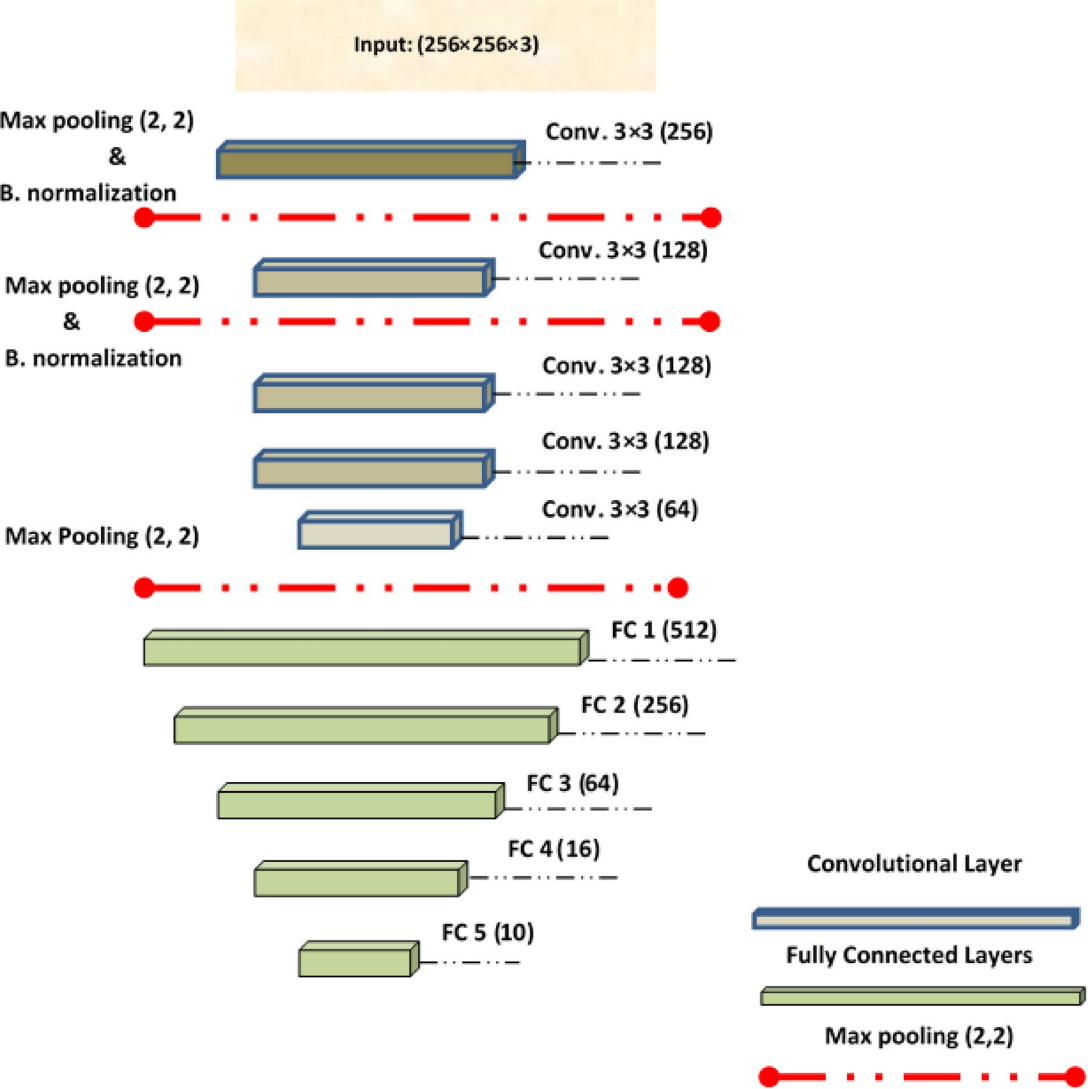
Complete structure of the proposed CNN architecture.

The development of a high-performance CNN-based deep learning model with minimal error and loss necessitates careful selection of several critical hyperparameters. These include the optimization algorithm, network architecture, filter dimensions, batch size, activation functions, learning rate, and the number of filters suited to the specific characteristics of the dataset. The proposed CNN architecture incorporates multiple convolutional layers interleaved with max-pooling operations, batch normalization, and fully connected (dense) layers. A deliberate and systematic approach to the layering, pairing, and integration of these components enables the construction of customized architecture designed to exceed the performance of standard pre-trained models. This design emphasizes modularity and efficiency, enabling the model to extract deep hierarchical features while maintaining computational feasibility. The complete structural layout of the proposed CNN algorithm is illustrated in Fig. 5.

### The proposed CNN architecture (Pyramidal Net)

The proposed CNN architecture, referred to as Pyramidal Net, begins with an input layer designed to accept RGB images with dimensions of (256, 256, 3). The architecture is inspired by a pyramidal structure, progressively reducing spatial dimensions while increasing feature abstraction, enabling robust object classification in satellite imagery. The core of the model consists of five convolutional layers, interspersed with three max-pooling layers and two batch normalization layers. All convolutional layers employ **ReLU** as the activation function and use ‘valid’ padding. The structure is detailed as follows:

1. **Conv Layer 1**: 256 filters, kernel size 3 × 3, ReLU activation.
2. **Max-Pooling Layer 1**: Kernel size 2 × 2, ‘valid’ padding.
3. **Batch Normalization 1**.
4. **Conv Layer 2**: 128 filters, kernel size 3 × 3, ReLU activation.
5. **Max-Pooling Layer 2**: Kernel size 2 × 2, ‘valid’ padding.
6. **Batch Normalization 2**.
7. **Conv Layer 3**: 128 filters, kernel size 3 × 3, ReLU activation.
8. **Conv Layer 4**: 128 filters, kernel size 3 × 3, ReLU activation.
9. **Conv Layer 5**: 64 filters, kernel size 3 × 3, ReLU activation.
10. **Max-Pooling Layer 3**: Kernel size 2 × 2, ‘valid’ padding.
11. **Flatten Layer**: Produces a 1D vector of size 50,176.
12. **Fully Connected Block**: Five dense layers with ReLU activation and units arranged in descending order: 512, 256, 64, 16, and 10.

**Fig. 6:**
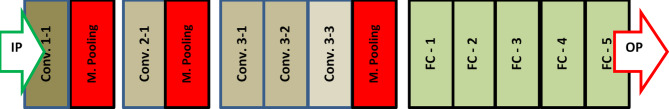
Overview of the proposed CNN architecture’s structure.

The output layer consists of a fully connected (dense) layer comprising 10 classes, employing the SoftMax activation function to align with the number of target classes. To compute the loss, sparse categorical cross-entropy is utilized, which is well-suited for multi-class classification tasks involving integer-encoded labels. Model optimization is performed using the Adam optimizer, with specific hyper parameters detailed in the Results section. This optimization approach, in conjunction with the pyramidal network architecture, facilitates superior feature extraction and classification performance relative to conventional pre-trained models. The model’s hierarchical structure, along with its filter dimensions and kernel configurations, collectively enhance both training efficiency and classification accuracy. Figure 6 presents the summarized architecture of the proposed CNN model.

## Experimental results and discussion

### Dataset description

To evaluate the performance of the proposed CNN model (Pyramidal Net), two benchmark remote sensing datasets were employed:

- **NWPU-RESISC45 Dataset**: A subset of this large-scale remote sensing dataset was used, consisting of 10 distinct classes (harbor, chaparral, tennis court, industrial area, parking lot, forest, beach, overpass, airplane, and baseball diamond) with 700 unique images per class.
- **UC Merced Land Use Dataset**: This dataset served as an additional validation source. Similarly, a subset of 10 selected classes was used (agricultural, airplane, baseball diamond, beach, building, chaparral, river, forest, freeway, and golf course) with 100 unique images per class.

To ensure a fair and consistent evaluation, ten random classes from each dataset were selected for training, validating and testing. For both datasets, the images were split into 70% for training, 15% for validating and 15% for testing, maintaining equal class distribution to prevent class imbalance. With details, the dataset was divided into training, validation, and testing subsets. Specifically, the training set comprises **5**,**057 RGB images**, each with a resolution of **256 × 256 pixels**, accompanied by their corresponding class labels. The validation set includes **893 images** of the same dimensions, while the test set contains **1**,**050 images**, all maintaining the consistent **three-channel (RGB)** format.

### Performance metrics

The performance of the proposed object classification model in remote sensing imagery was assessed using several evaluation metrics. These metrics consider classification accuracy, computational efficiency, and model complexity.

#### Key metrics include

- **Accuracy**: Defined as the proportion of correctly classified instances over the total number of predictions made. It is calculated as:

1 $$\mathrm{Accuracy}\:=\:\frac{\mathrm{T}\mathrm{N}+\mathrm{T}\mathrm{P}\:}{\mathrm{T}\mathrm{N}+\mathrm{T}\mathrm{P}+\mathrm{F}\mathrm{N}+\mathrm{F}\mathrm{P}}$$

- **The precision** of an algorithm is determined by dividing its total number of true positive classifications by its total number of positive classifications.

 

2 $$\mathrm{Precision}\:=\:\:\frac{\mathrm{T}\mathrm{P}\:}{\mathrm{T}\mathrm{P}+\mathrm{F}\mathrm{P}}$$

 

- **A measure of recall** is the ratio of real positively classified samples to all positively classified samples.

 

3 $$\mathrm{Recall}\:=\:\frac{\mathrm{T}\mathrm{P}\:}{\mathrm{T}\mathrm{P}+\mathrm{F}\mathrm{N}}$$

- **The F1 score** is the weighted mean of precision and recall.

 

4 $$\text{F1 score}\: =\: 2 \times\:\frac{\mathrm{precision}\times\:\:\mathrm{recall}\:\:}{\mathrm{precision}\:+\:\mathrm{recall}\:}$$

- The similar metric is called Intersection Over Union (IOU). Additionally, it is also known as Jaccard and represents the ratio of all negative classifications to the entire number of actual positive categories.

 

5 $$\mathrm{IOU}\:=\:\frac{\mathrm{T}\mathrm{P}\:}{\mathrm{T}\mathrm{P}+\mathrm{F}\mathrm{N}+\mathrm{F}\mathrm{P}}$$

### Parameters

The training and architecture of the proposed CNN model were governed by a set of well-defined hyperparameters that ensured consistency, performance, and fairness in experimentation. Table 4 summarizes the training and optimization parameters used throughout the study. The model was trained using the Adam optimizer with an initial learning rate of 0.00025 and a ReduceLROnPlateau scheduler, which adaptively reduced the learning rate by a factor of 0.25 if validation loss plateaued over four consecutive epochs. The training strategy followed a 70/15/15 split for training, validation, and testing, respectively, with a batch size of 8 and a fixed random seed (32) to ensure reproducibility. Sparse categorical cross-entropy was employed as the loss function, suitable for multi-class classification tasks with integer labels.

Table 5 presents the architectural and data-specific hyperparameters of the proposed pyramidal CNN. The network consisted of five convolutional layers with progressively decreasing filter counts, interspersed with max-pooling and batch normalization layers to control feature dimensionality and training stability. This was followed by a sequence of fully connected layers culminating in a 10-unit softmax output for classification. The input images were resized to 256 × 256 × 3, and several data augmentation techniques were applied to enhance robustness, including horizontal and vertical flipping, random rotations between 0° and 180°, and brightness variation in the range [0.1–2.0]. These preprocessing strategies collectively improved the model’s generalization capacity and resistance to overfitting, particularly in the context of remote sensing image variability.

**Table 4:** Training and optimization hyperparameters.

Category	Hyperparameter	Value/Description
Training Strategy	Train/Validation/Test Split	70%/15%/15%
	Epochs	30
	Batch Size	8
	Random Seed	32
Optimizer	Optimizer	Adam
	Initial Learning Rate	0.00025
	Minimum Learning Rate	1 × 10⁻⁶
	LR Scheduler	ReduceLROnPlateau
	LR Reduction Factor	0.25
	Patience (before LR reduction)	4 epochs
	Weight Decay	adding an L2 norm term to the loss function.
	Dropout Rate	-
	Kernel Size	(3 × 3)
	Stride	One step
	Padding	(“valid”)
	Activation Function	ReLU
	Data Normalization	Only resizes to (256,256)
Loss Function	Type	Sparse Categorical Cross entropy

**Table 5:** CNN architecture and input/preprocessing hyperparameters.

Category	Hyperparameter	Value/Description
CNN Architecture	Conv Layer 1	256 filters, 3 × 3 kernel, ReLU
	Conv Layer 2	128 filters, 3 × 3 kernel, ReLU
	Conv Layer 3	128 filters, 3 × 3 kernel, ReLU
	Conv Layer 4	128 filters, 3 × 3 kernel, ReLU
	Conv Layer 5	64 filters, 3 × 3 kernel, ReLU
	Pooling	MaxPooling 2 × 2,‘valid’ padding (after Conv1, Conv2, Conv5)
	Batch Normalization	Applied after Conv1 and Conv2
	Fully Connected Layers	512 → 256 → 64 → 16 → 10 (Softmax at output)
Input Dimensions	Image Size	256 × 256 × 3
Data Augmentation	Rotation Range	0°–180°
	Horizontal Flip	Enabled
	Vertical Flip	Enabled
	Zoom Range	0.0 (disabled)
	Brightness Range	[0.1–2.0]

### Selection of optimization parameters

This section discusses the rationale behind the selection of key optimization parameters and analyzes their impact on the classification accuracy of the proposed deep learning model for object recognition in remote sensing imagery. A critical choice in the model’s training configuration was the use of the Adam optimizer, known for its adaptive learning rate and efficient convergence properties. Adam was selected due to its robustness, minimal need for hyperparameter tuning, and consistent performance across various deep learning tasks. This decision was supported by both theoretical justification and empirical evidence from related studies. To evaluate the impact of the optimizer on classification performance, a series of experiments were conducted using various combinations of initial learning rates and batch sizes. The learning rates assessed ranged from 1 × 10⁻⁶ to 0.001. It was observed that very low learning rates considerably increased convergence time without yielding substantial improvements in accuracy, while higher learning rates resulted in unstable convergence and signs of overfitting. An initial learning rate of 0.00025 was identified as providing the optimal balance between convergence efficiency and model generalization. Alongside learning rate, batch size was also examined as a critical hyperparameter during training, with values of 8, 16, 32, 50, and 64 systematically evaluated. Larger batch sizes generally reduced training time but were prone to converge to suboptimal local minima. In contrast, smaller batch sizes improved generalization but occasionally introduced overfitting. The batch size of 8 produced the most favorable results in terms of both accuracy and training stability, offering a strong balance between convergence efficiency and model performance.

### Experiments

This study evaluates and compares the performance of the proposed CNN model, Pyramidal Net, against five widely recognized pre-trained convolutional neural networks (CNNs) for object recognition in remote sensing imagery. The comparison is based on multiple performance metrics, including accuracy, precision, recall, F1-score, and Intersection over Union (IOU). The experimental workflow begins with image preprocessing, which includes resizing to fit the input dimensions required by each model, followed by data augmentation to mitigate overfitting and enhance generalization. The same augmentation strategy is applied across all models to ensure consistency. The following five pre-trained CNN architectures are selected for benchmarking: VGG19, VGG16, Mobile Net, ResNet50, and Xception.

All models, including the proposed architecture, are trained and evaluated using the same datasets outlined in Sect. “Dataset description”. To ensure a fair and consistent comparison, training is conducted over a fixed duration of 30 epochs under identical hardware and software conditions. Following the training phase, each model undergoes testing, during which the defined performance metrics are calculated. These results serve as the basis for assessing the effectiveness of each model in performing object classification tasks within the domain of remote sensing. Table 6 provides a summary of the input image dimensions required for each model to facilitate reproducibility and highlight architectural differences in input constraints.

**Table 6:** Input image dimensions for the proposed CNN algorithms.

Algorithms	Input resizes
VGG – 19	(224,224,3)
VGG – 16	(224,224,3)
Mobile-Net	(224,224,3)
Res-Net 50	(224,224,3)
Xception	(299,299,3)
Proposed model	(256,256,3)

The article utilized the default values provided by the Keras library for each model. We tested the proposed model with an input size of 256 × 256 × 3, which did not affect performance but did extend the training duration. This was despite one of the objectives of the proposed model being to enhance accuracy while reducing training time compared to other models. The three phases of the object detection process are thoroughly outlined in Algorithm 1 for the proposed model.

**Figure:**
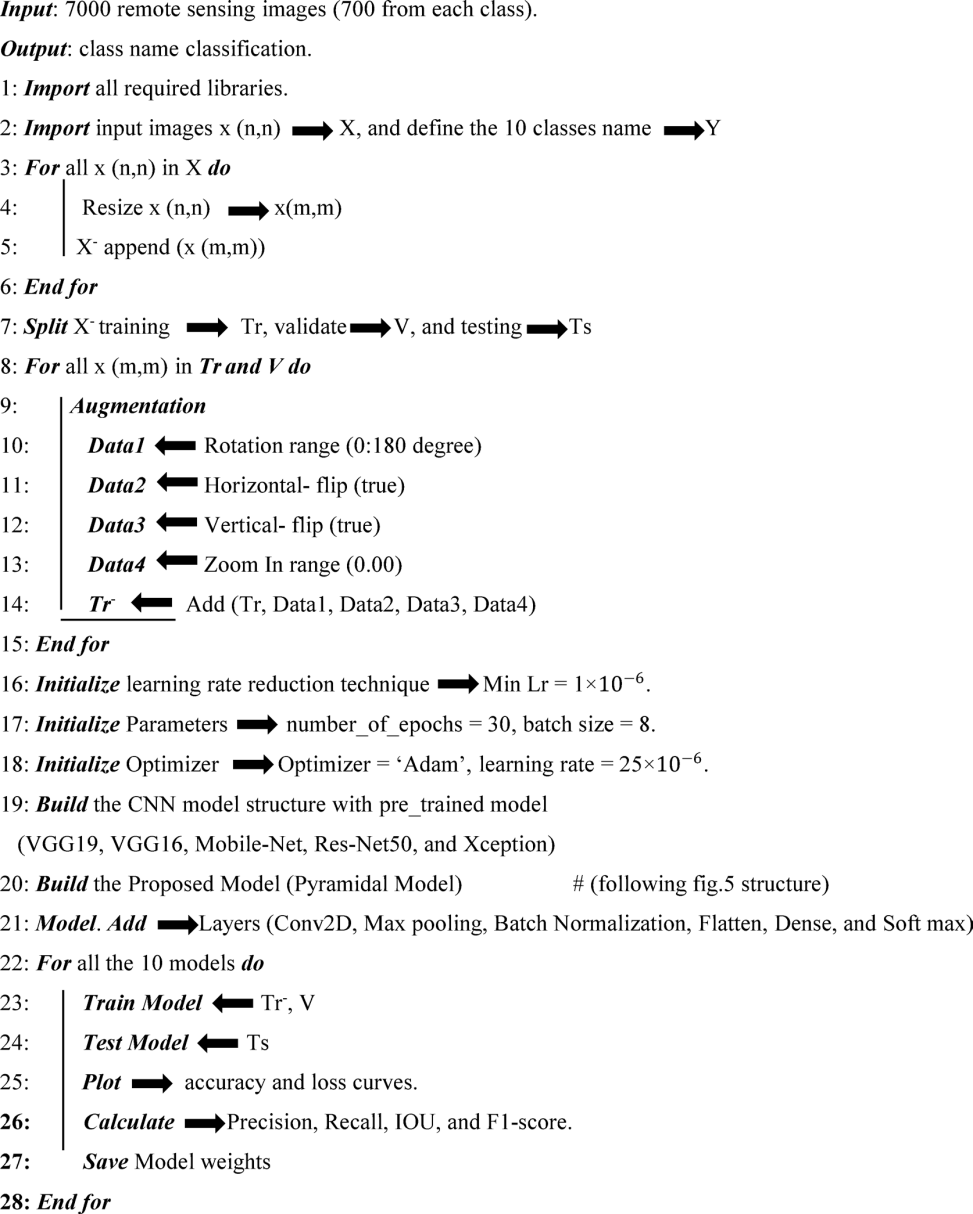
**Algorithm 1:** The object detection method proposed.

**Fig. 7:**
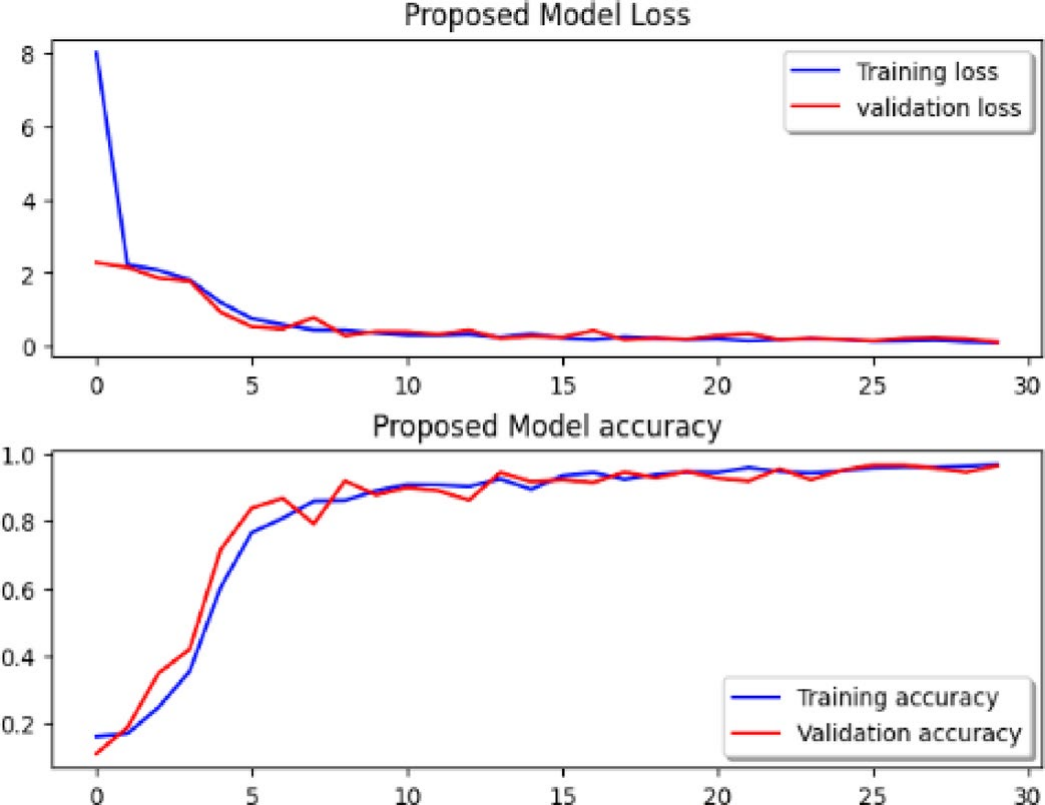
VGG-19 loss curve, VGG-19 accuracy curve.

**Fig. 8:**
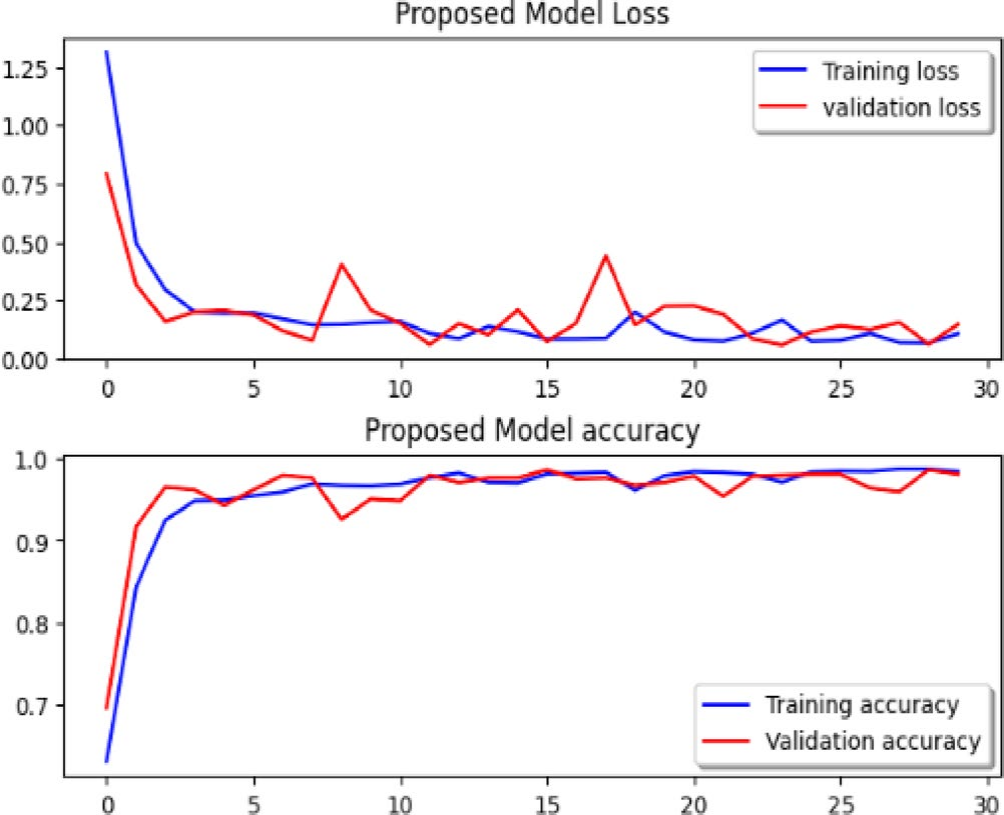
VGG-16 loss curve, VGG-16 accuracy curve.

**Fig. 9:**
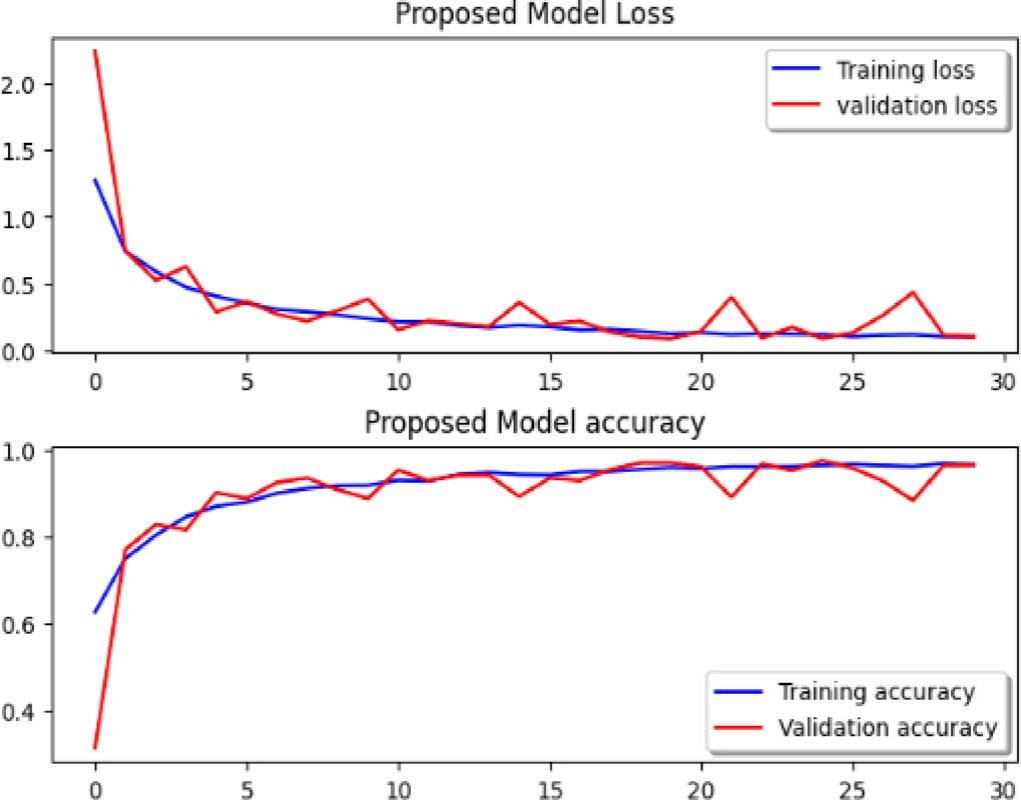
Xception loss curve, Xception accuracy curve.

**Fig. 10:**
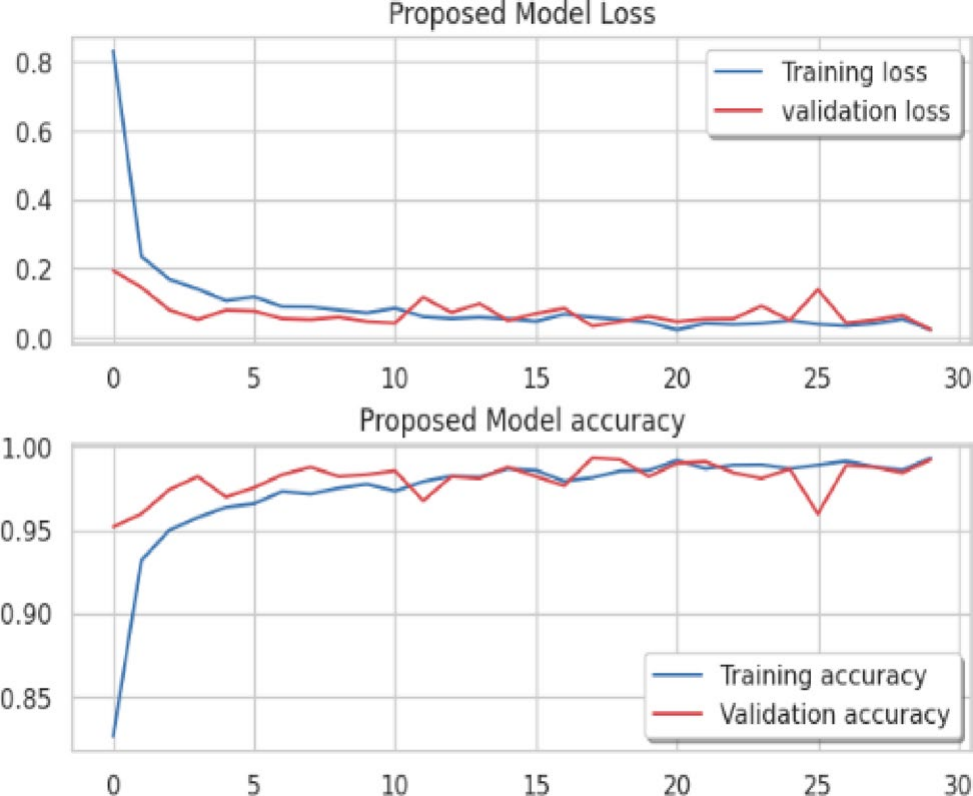
Mobile-Net loss curve, Mobile-Net accuracy curve.

**Fig. 11:**
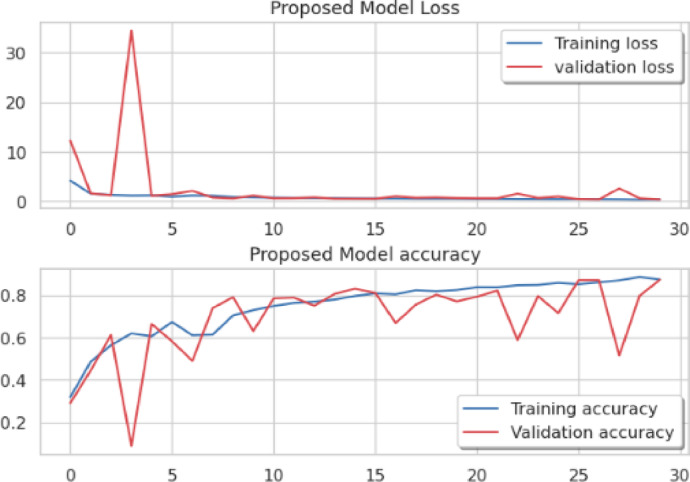
Res-Net50 loss curve, Res-Net152V2 accuracy curve.

**Fig. 12:**
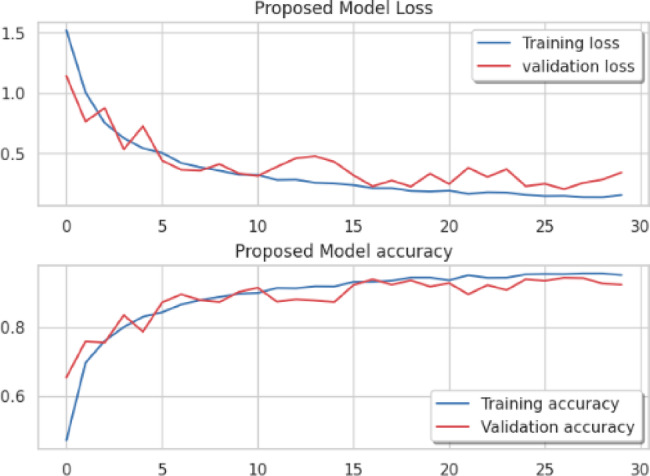
Proposed model loss curve, proposed model accuracy.

**Fig. 13:**
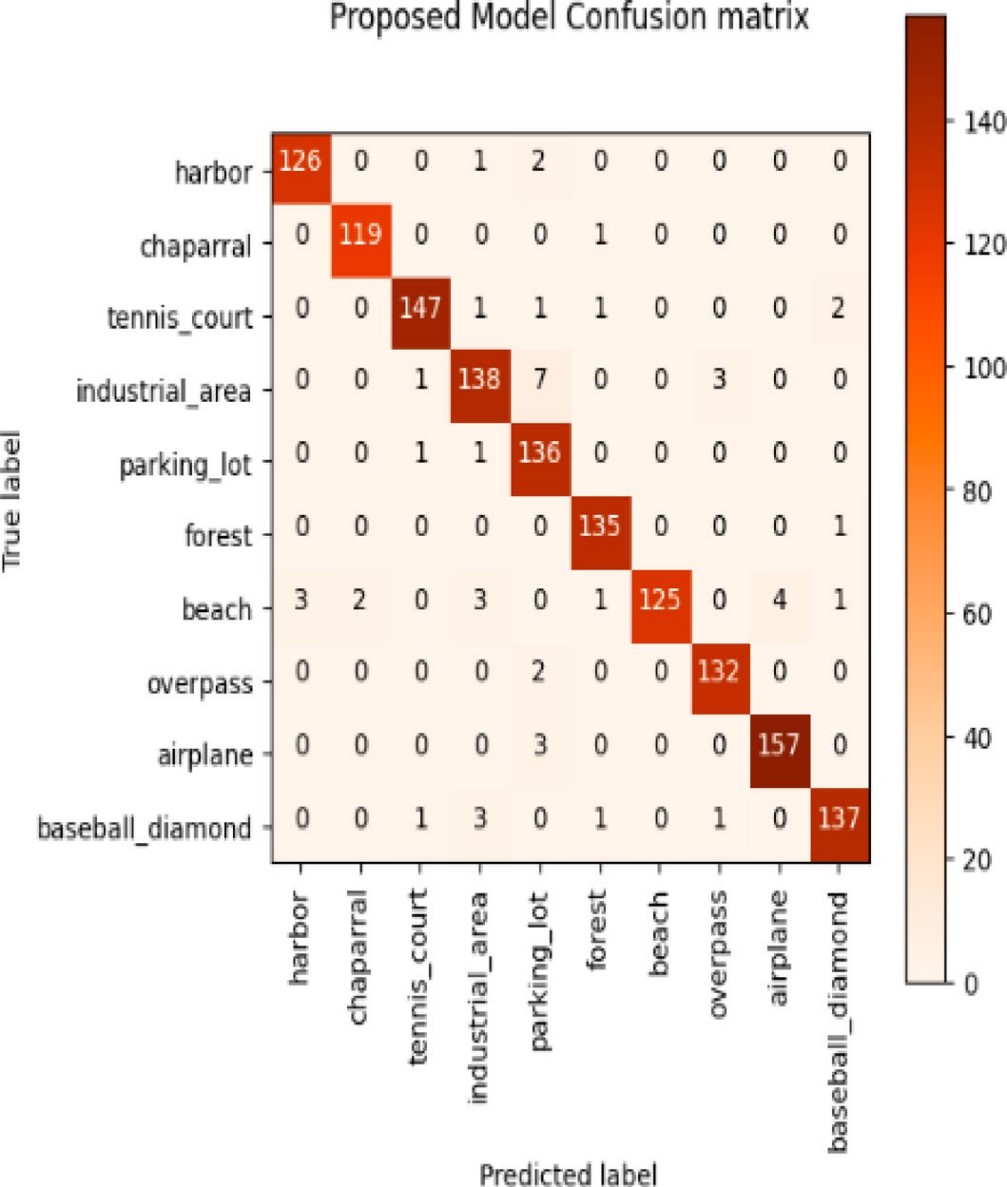
VGG19 confusion matrix.

**Fig. 14:**
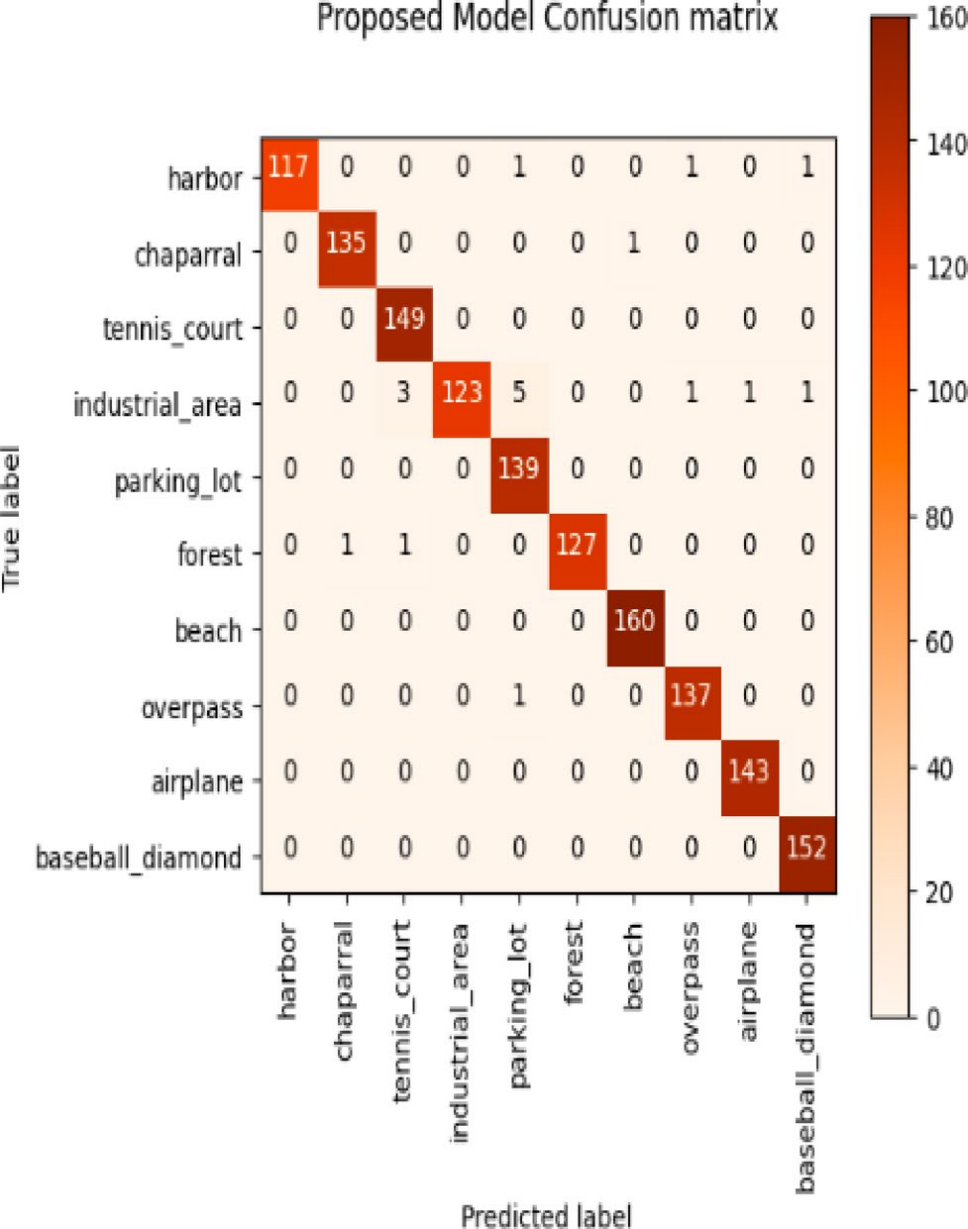
VGG16 confusion matrix.

**Fig. 15:**
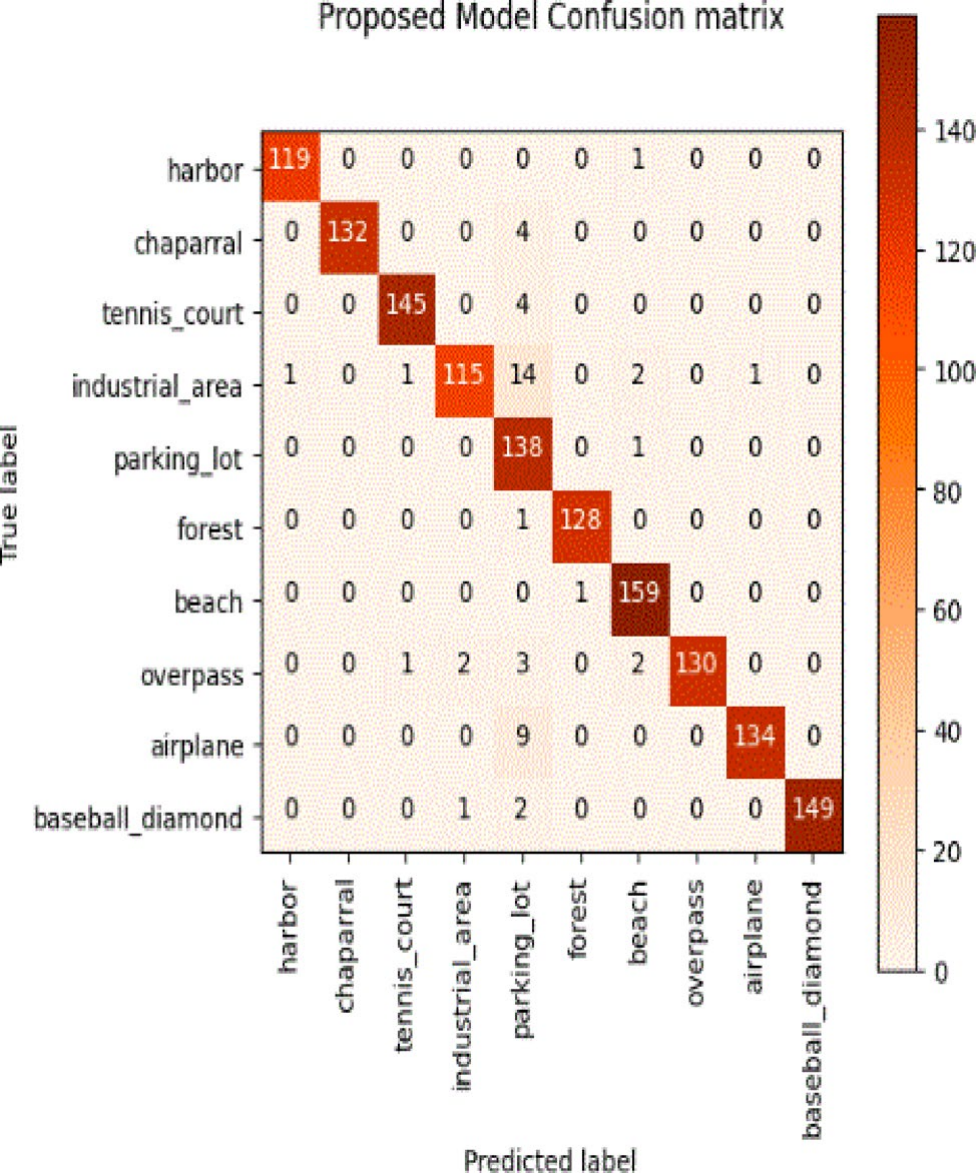
Xception confusion matrix.

**Fig. 16:**
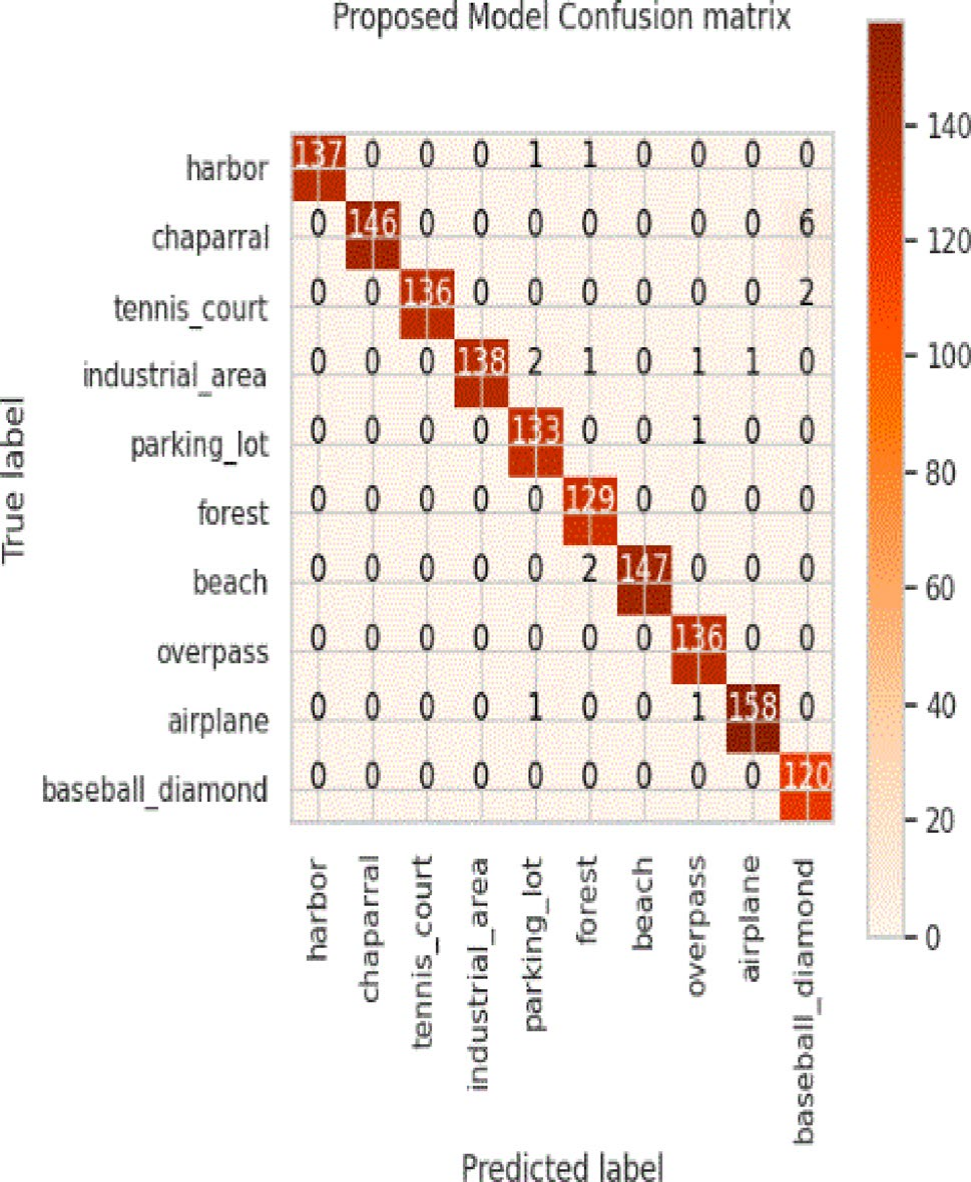
Mobile-Net confusion matrix.

**Fig. 17:**
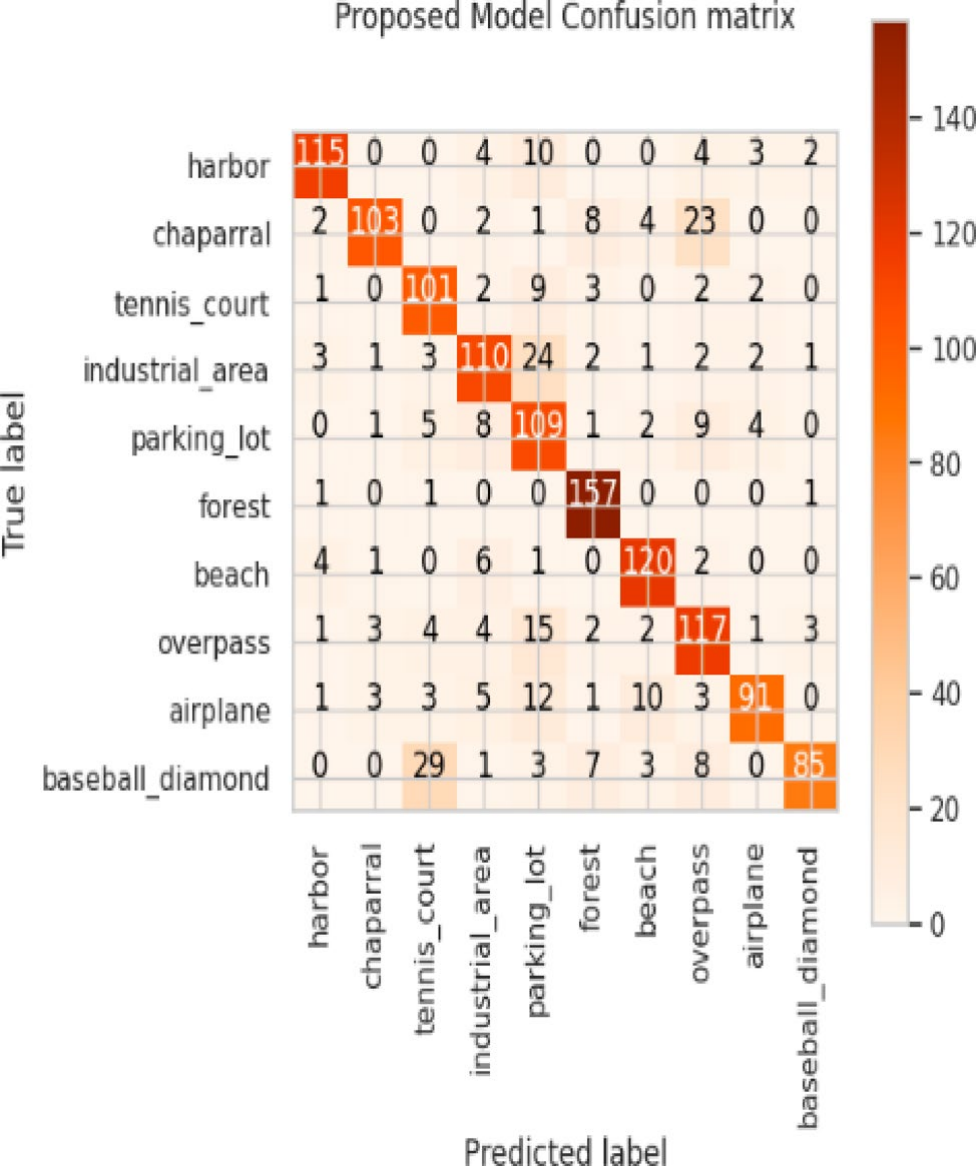
Res-Net50 confusion matrix.

**Fig. 18:**
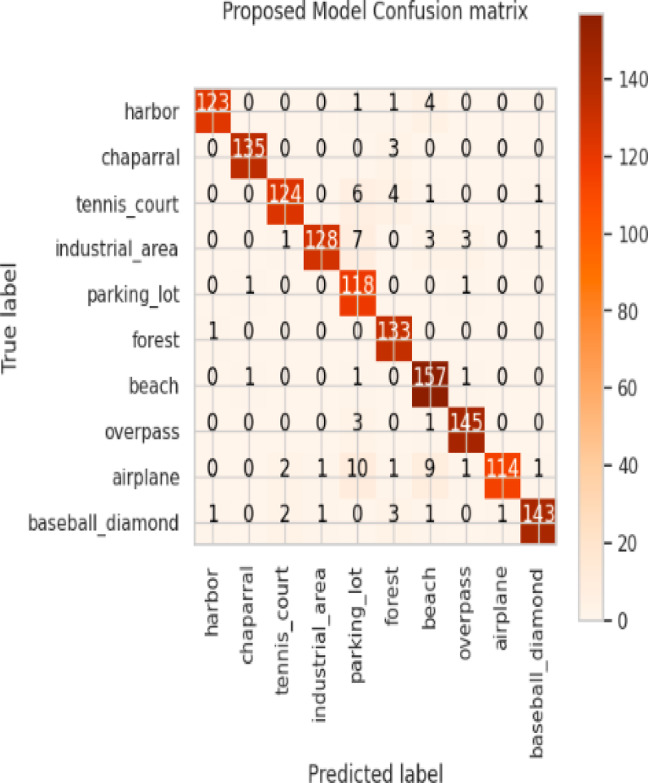
Proposed model confusion matrix.

Considering a uniform training regime of 30 epochs across all models, Table 7 presents a detailed comparative analysis between the proposed CNN model and several state-of-the-art pre-trained CNN architectures. The comparison includes key performance indicators: training loss, training accuracy, test loss, and test accuracy. To provide further insight into the model behavior during training, Figs. 7, 8, 9, 10, 11 and 12 illustrates the accuracy and loss curves for both the training and validation phases. These visualizations aid in understanding convergence behavior, overfitting tendencies, and model stability. Moreover, Figs. 13, 14, 15, 16, 17 and 18 display the confusion matrices for each model, offering a class-wise performance evaluation and highlighting the strengths and weaknesses in multi-class object recognition. The empirical results indicate that the proposed CNN model consistently outperforms all evaluated pre-trained architectures with respect to accuracy and generalization across both datasets. While models such as Xception and VGG16 demonstrate competitive performance, the Pyramidal Net achieves the highest accuracy and the shortest training time, thereby providing an optimal balance between computational efficiency and predictive effectiveness.

**Table 7:** A comparison of various pre-trained CNN algorithms with the proposed model on the NWPU-RESISC45 dataset.

CNN model	Test loss	Test accuracy	Train loss	Train accuracy	Epochs	Precision	Recall	F1-score	IOU	Image size	Training time/Model speed
VGG 16	0.188	0.949	0.073	0.975	30	0.95	0.95	0.95	0.902	(224,224,3)	2303.5
VGG 19	0.407	0.858	0.350	0.881	30	0.86	0.86	0.86	0.751	(224,224,3)	2360.1
Mobile Net	0.558	0.810	0.488	0.825	30	0.82	0.81	0.81	0.680	(224,224,3)	1995.6
ResNet 50	0.415	0.865	0.363	0.886	30	0.87	0.86	0.87	0.762	(224,224,3)	3454.5
Xception	0.168	0.948	0.060	0.980	30	0.95	0.95	0.95	0.900	(299,299,3)	4749.9
Proposed model	0.19	0.9428	0.131	0.957	30	0.95	0.94	0.94	0.89	(256,256,3)	3692

Both the pre-trained algorithms and the suggested CNN method have been applied to the UC Merced Land Use datasets in order to guarantee the experimental outcome findings. The experimental findings demonstrated that the proposed pyramidal model performs better than every other CNN model that has been assessed.

Additionally, as Table 8 illustrates, the pyramidal model performs exceptionally well on tiny datasets in contrast to the majority of pre-trained models, which suffer from overfitting.

**Table 8:** Comparison of various pre-trained CNN algorithms with the proposed model on the UC Merced land use dataset.

CNN model	Test loss	Test accuracy	Train loss	Train accuracy	Epochs	Precision	Recall	F1-score	IOU	Image size	Training time/Model speed
VGG 16	1.14	0.667	0.920	0.673	30	0.61	0.67	0.63	0.50	(224, 224,3)	453.6
VGG 19	1.27	0.567	0.962	0.633	30	0.50	0.57	0.50	0.39	(224, 224,3)	421.0
Mobile Net	3.29	0.100	1.315	0.557	30	0.01	0.10	0.02	0.05	(224, 224,3)	124.0
ResNet 50	0.89	0.707	0.476	0.857	30	0.74	0.71	0.70	0.54	(224, 224,3)	502.2
Xception	2.30	0.100	0.075	0.971	30	0.01	0.10	0.02	0.05	(299, 299,3)	266.1
Proposed model	0.34	0.93	0.25	0.93	30	0.94	0.93	0.93	0.86	(256,256,3)	559

An extra experiment was performed to evaluate the accuracy based on the size of the output classes, ensuring that the proposed model is size-invariant and can classify objects of different sizes. The experiment used five classes from the NWPU-RESISC45 dataset: small-sized objects (harbor), medium-sized objects (beach), and 700 images from five objects. After that, these 3500 photos are divided into a 70% training set, 15% validate set and a 15% test set.

The performance metrics of the suggested model throughout this experiment are displayed in Tables 9 and 10.

**Table 9:** Presents the performance metrics of the proposed model for each five classes in the dataset individually.

Classes	Precision	Recall	F1- score
Harbor	0.98	0.95	0.97
Chaparral	0.99	0.98	0.98
tennis court	0.96	0.91	0.94
Forest	0.92	0.99	0.95
Beach	0.89	0.98	0.93

**Table 10:** Displays the overall performance of the proposed model on the 5-class dataset.

CNN structure model	Train accuracy	Train loss	Test accuracy	Test loss	Epochs	Batch size
Our Proposed model results	0.951	0.19	0.9428	0.12	30	8

**Fig. 19:**
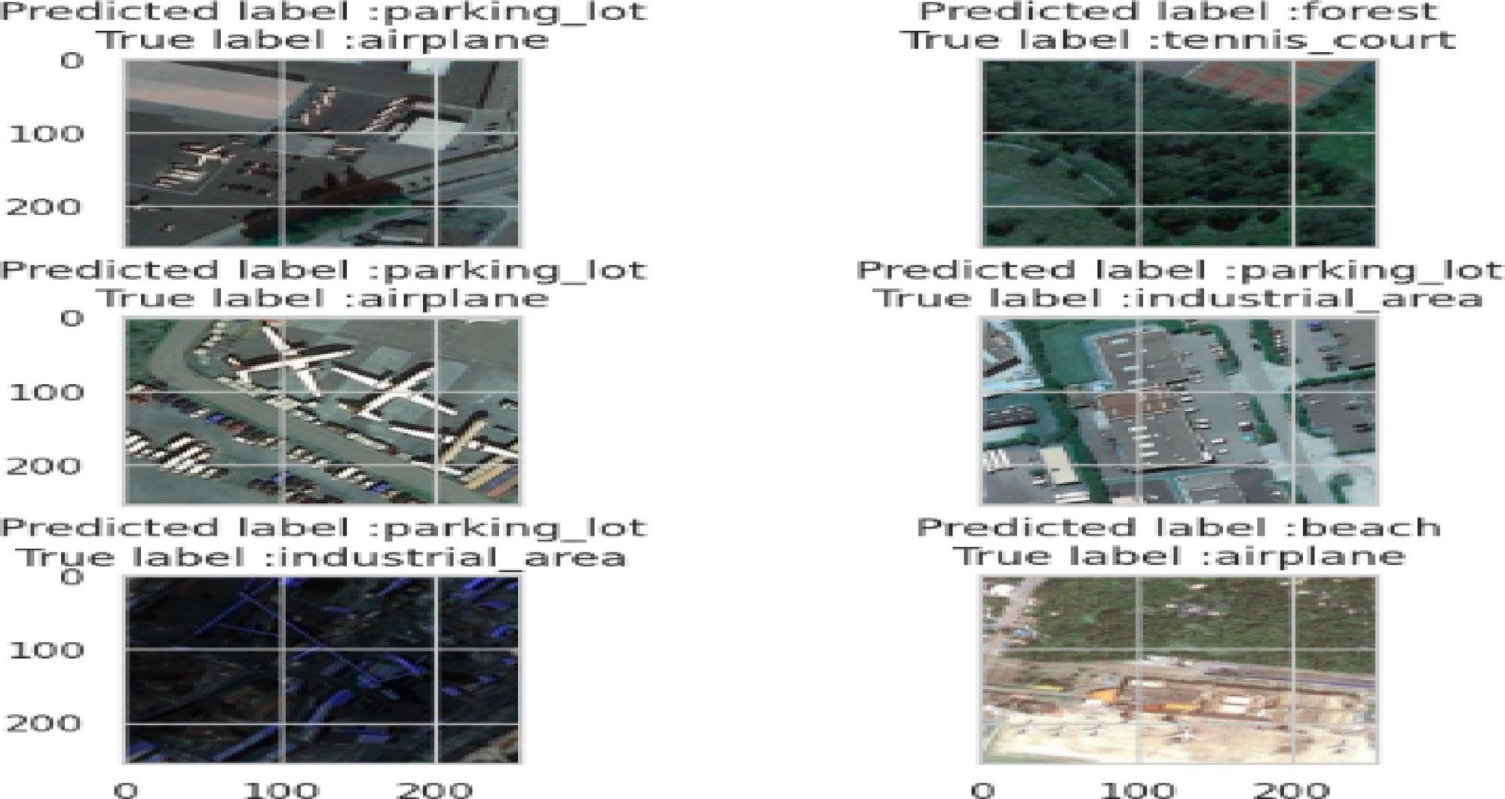
Errors are difference between predicted labels and true labels.

Figure 19 visualizes the errors between the predicted and actual class labels made by the proposed CNN model. This error plot helps highlight specific instances where the model’s classification does not align with the ground truth, offering insights into potential class confusions. Such visualization is valuable for diagnosing model limitations, particularly in visually similar scene classes or under challenging conditions such as low contrast or partial occlusion.

In order to improve the model’s capacity to adapt to changing lighting conditions, brightness augmentation with a brightness range of [0.1:2] was employed during dataset compilation.

The model’s resilience in a range of lighting conditions is increased by this augmentation technique.

The number of convolutional layers in the suggested model’s filters is split by three in this experiment to prevent overfitting. Lastly, the paper compared the suggested approach with a few recently released research articles in the field of remote sensing image classification, which are shown in Table 11, to ensure that the suggested model has a favourable impact on such remote sensing image classification performance.

**Table 11:** Presents a comparison of the proposed method with related works in terms of classification.

Model	Year	Accuracy	Recall	F1-score	Training time	Complexity
ViT-Base with ResNet50 (Seddik Boudissa.et al.2024)^46^	2024	87.45	-	87.1	High	High
Vision Transformer via MapReduce (Rondi Pushpa Latha, and Persis Voola.,2025)^47^	2025	92	-	96	Low	Low
Hybrid-Model KD ‐HMKD Net comprises a CNN‐ViT (Huaxiang Song, YuxuanYuan, Zhiwei Ouyang, YuYang, and HuiXiang.,2024)^48^	2024	96.44	96.43	96.43	High	High
Deep Learning-Based Vision Transformer (Adekanmi Adegun, Serestina Viriri, and Jules-Raymond Tapamo.,2024)^49^	2024	96	90	94	High	Medium
Global–Local Dual-Branch Structure Model (Xu et al., 2022)^50^	2022	94.46	93.2	93.1	Medium	High
mms CNN–HMM combined model with stacking ensemble (Cheng, and Lei, 2022)^51^	2022	95.5	-	-	High	High
Two-Speed Deep-Learning ensemble (Horry et al., 2023)^52^	2023	65	64	63	Low	Low
Embedded Linear Vision Transformer ELVIT (Yunfei Tan et al.,2025)^53^	2025	98.43	99.67	99.27	Low	Medium
LEViT(Yihan Chen et al.,2022)^54^	2022	91.56	91.56	91.45	Low	Low
fine-tuning the ResNet-50 (Muhammad Akhtar et al.,2023)^55^	2023	99.5	99.5	99.5	High	High
Proposed model	2025	94.28	94	94	Low	Low

## Interpretability structure

### Interpretability methods

To enhance the transparency of our model, we incorporated two complementary interpretability techniques: Shapley Additive Explanations (SHAP) for global feature attribution and Gradient-weighted Class Activation Mapping (Grad-CAM) for local visual explanations. SHAP provides insight into the contribution of input features to the model’s predictions across the entire dataset, while Grad-CAM highlights spatially significant regions within individual test images. The combined use of these methods allows for a more holistic understanding of the model’s decision-making process.

For quantitative validation, we performed a pixel-wise overlap analysis between the normalized SHAP heatmaps and Grad-CAM activation maps. The overlap was computed as the percentage of intersecting salient pixels above a predefined threshold, providing a measure of alignment between global and local interpretability cues.

### Quantitative interpretability results

The pixel-wise overlap analysis revealed a mean correspondence of 85% between SHAP and Grad-CAM outputs across the test set. This substantial agreement indicates that the most influential features globally (e.g., linear runways, water bodies, vegetated fields) also manifest as spatially dominant in local visualizations. These findings support the consistency of our interpretability framework and its ability to highlight meaningful patterns in remote sensing scenes.

Examples from key land use classes (e.g., Airport, Harbor, Baseball Diamond) demonstrate how SHAP values prioritize domain-relevant features, such as long, narrow regions for runways or high green pixel density for recreational fields, which are also emphasized by Grad-CAM maps.

### Importance of interpretability in remote sensing

Interpretability is still a rare but increasingly important aspect of deep learning in remote sensing, especially within recent Vision Transformer-based models, where global explanation tools like SHAP are scarcely applied. By integrating SHAP with Grad-CAM, our model not only maintains competitive classification performance but also provides transparent and explainable predictions—an essential quality in applications where decisions have operational or environmental implications.

The observed 85% overlap between SHAP and Grad-CAM maps reinforces the trustworthiness of our model’s explanations, allowing domain experts to validate outcomes and uncover data-driven insights. For instance, the model’s ability to associate specific spatial patterns (e.g., water pixels in harbors or compact geometric layouts in baseball diamonds) with class predictions can aid in monitoring, planning, or change detection tasks.

## Aspects contributed to accuracy improvement

The study presented various configurations—including the number of filters, layer architecture, kernel size, and optimization strategies aimed at enhancing the performance of the proposed model. The model’s distinctive and optimized layer arrangement contributes significantly to improved feature extraction and classification accuracy, distinguishing it from conventional architecture, which includes carefully chosen filter numbers, kernel sizes, and overall hierarchical representation, enhanced feature extraction, and boosted system performance. A learning rate reduction strategy was also employed to improve model generalization and speed up convergence. Moreover, data augmentation techniques were applied, increasing the model’s exposure to various data variations and strengthening its robustness. Adam was later selected as the optimization method that enhanced the utilization of gradient data and accelerated convergence.

### Challenges

The primary potential difficulties that the suggested model might run into have been emphasized in this section:

1. **Limited data set variation**: The absence of a dataset with fluctuations in illumination, surroundings, and seasonal circumstances is one of the model’s shortcomings.
2. **Labelling and annotation errors**: The quality of the labels used for the training data has a significant impact on the suggested model’s accuracy. Inherent errors in labelling may have an impact on the overall model’s performance.
3. **Transferability to other domains**: Our model may require extra fine-tuning or modifications to be applied to other datasets or domains, even if it is intended for object categorization in satellite images.
4. **Temporal consistency**: Our model could not be tested because the datasets are single snapshot, we plan to extend our approach to multi-temporal datasets to assess the temporal stability and consistency of the model’s predictions.
5. **Limitations and Corresponding Solutions for Severe Occlusion** (e.g., clouds, shadows, buildings blocking view):

**Solution**:

1. Employ data augmentation techniques that simulate occlusions during training (e.g., CutMix, random erasing).
2. Incorporate attention mechanisms or transformer-based modules to help the model focus on unobstructed regions.
3. Use multi-view or multi-temporal data fusion (e.g., combining images from different angles or times) to compensate for occluded areas.

**Extreme Image Noise** (e.g., sensor artifacts, low resolution):

**Solution**:

4. Preprocess input images with denoising filters or autoencoders.
5. Train the model using noisy image augmentation to improve robustness.
6. Explore robust loss functions that reduce the impact of noisy or mislabeled pixels.

**Dramatic Seasonal Variations** (e.g., snow in winter, dry terrain in summer):

**Solution**:

7. Include seasonally diverse training data to improve generalization.
8. Use domain adaptation techniques to minimize the distribution shift between seasons.
9. Integrate temporal metadata (e.g., time of capture) as auxiliary inputs.

**Unseen Classes** (Zero-shot or few-shot classification):

**Solution**:

10. Apply few-shot learning or meta-learning approaches to adapt to new classes with limited labeled samples.
11. Use semantic embeddings (e.g., word vectors) for class labels to enable zero-shot classification.
12. Implement self-supervised pretraining to enhance the model’s general understanding of image structure.

**Minimal labeled data in new domains:**

**Solution:**

13. Use transfer learning from large, annotated datasets to smaller, domain-specific ones.
14. Explore semi-supervised or self-supervised learning methods to exploit unlabeled data.

### Areas of improvement

1. Improve data augmentation: employ a wider range of intricate data augmentation approaches to increase the model’s resilience to changes in satellite imagery, including fluctuations in lighting, weather, and seasonal conditions, Although the dataset used for training was captured in summer and testing was conducted on a different set of images taken in winter, these images still require more precise processing to yield more accurate results.
2. Preprocessing: Develop preprocessing techniques that enhance noise reduction, feature extraction, and general quality before adding satellite photos to models.
3. Object localization: In addition to classifying objects, enhance the model’s ability to precisely locate and define object borders inside the satellite images.
4. Ensemble Models: To increase the overall classification accuracy, experiment with ensemble learning strategies by combining various models or model variations.

## CNN model contribution

The proposed CNN model offers a well-optimized balance between classification accuracy, computational efficiency, and generalization capability, outperforming or equaling several deep pre-trained architectures across two widely used remote sensing datasets. On the NWPU-RESISC45 dataset, the model achieves a test accuracy of 94.28%, which is comparable to models such as VGG16 and Xception, while employing a lighter architecture with faster training time and reduced overfitting, as evidenced by a minimal gap between training and testing losses.

On the UC Merced Land Use dataset, which presents challenges due to its smaller size and class imbalance, the proposed model demonstrates exceptional generalization with a test accuracy of 93% and a substantial improvement over several pre-trained models, some of which perform as low as 10% accuracy on this dataset.

The model consistently achieves high performance across various evaluation metrics, including precision, recall, F1-score, and intersection over union (IoU), with an IoU of 0.86 on the UC Merced dataset and 0.89 on the NWPU dataset, indicating reliable and balanced predictions across classes.

In addition to its accuracy, the model utilizes a moderate input resolution (256 × 256) and achieves competitive training time, making it well-suited for deployment in practical remote sensing scenarios that may involve resource constraints or real-time processing requirements.

Unlike recent approaches dominated by large-scale transformer-based or CNN–ViT hybrid models, our proposed architecture achieves a competitive accuracy of 94.28% while maintaining a significantly lower computational footprint, using approximately 50% fewer parameters than Swin-Small and nearly one-third the FLOPs of ResV2ViT. This makes it a practical and scalable solution for applications requiring real-time performance or deployment on resource-constrained platforms.

A key innovation of this work is the integration of two complementary interpretability techniques SHAP (Shapley Additive Explanations) for global feature importance and Grad-CAM for spatial class activation visualization.

To the best of our knowledge, this is among the few works in recent literature that apply such a combined interpretability framework to remote sensing classification tasks. Our quantitative overlap analysis, showing up to 85% pixel-wise agreement between SHAP and Grad-CAM maps, provides compelling evidence of model transparency, which is critically important for applications in land use analysis, urban monitoring, and environmental assessment.

Furthermore, the proposed methodology emphasizes fair and consistent benchmarking. All models are trained and evaluated using the same 10-class subset, standardized train/validation/test splits, and identical preprocessing and augmentation pipelines. This contrasts with prior studies that report higher accuracy on larger class sets or smaller test sets, often leading to inflated performance estimates.

Lastly, while current datasets are temporally static, we incorporate seasonal augmentation strategies and propose future cross-season experiments to address generalization across temporal shifts an essential challenge in remote sensing imagery.

In summary, our contributions lie in presenting a balanced trade-off between accuracy, efficiency, generalization, interpretability, setting a precedent for transparent, lightweight, and context-aware scene classification models.

## Future work

A promising direction for future work involves integrating the **Vision Transformer (ViT)** architecture into the model pipeline. While convolutional neural networks (CNNs) have demonstrated strong spatial feature extraction capabilities, ViTs offer a powerful alternative by capturing global contextual relationships through self-attention mechanisms. This characteristic is particularly valuable in remote sensing, where spatial dependencies can extend over large image regions. Although current ViT-based models often require large-scale datasets and high computational resources, recent advancements in lightweight and hybrid ViT-CNN architectures present an opportunity for improved accuracy with manageable complexity. Also, the proposed model demonstrates strong performance across two benchmark remote sensing datasets. A key limitation of the current study is the inability to assess **temporal consistency**. This is because both datasets used NWPU-RESISC45 and UC Merced are composed of static, single-snapshot images, which do not provide multi-temporal observations of the same scenes. In real-world remote sensing applications, particularly those involving land-use monitoring, environmental change detection, and disaster assessment, the temporal stability of model predictions is crucial. Therefore, a natural and important direction for future research is to extend the proposed architecture to handle multi-temporal datasets. This will enable the evaluation of the model’s robustness over time and its ability to maintain consistent predictions under varying conditions such as seasonal changes, lighting differences, and anthropogenic activity.

## Conclusion

This study introduces a robust CNN model structure for object detection in remote sensing images. The NWPU-RESISC45 and UC Merced Land Use datasets are used to evaluate the effectiveness of the proposed models. The results demonstrate that the suggested pyramidal CNN model structure achieves the highest detection accuracy and the shortest training time when compared to well-established pre-trained CNN models. It is also highly effective for object detection tasks in remote sensing images, achieving an accuracy of up to 94.28%. The suggested model performed admirably when applied to various illumination datasets and object classes of varying sizes. Furthermore, in contrast to conventional pre-trained datasets, the pyramidal model demonstrated excellent performance while working with small datasets. More optimisation techniques, deep learning models, and classes might be used in future research. Furthermore, we might be interested in enhancing the suggested CNN structure in the future in addition to Future research will explore solutions to known limitations. For example, multi-temporal data fusion and attention-based modules can help overcome occlusion and seasonal variability, while noise-robust training and denoising preprocessing may address sensor-related noise. Furthermore, techniques such as few-shot learning, domain adaptation, and self-supervised pretraining will be considered to improve the model.

## Data Availability

The data that support the findings of this study are available within this article.
